# Clinical application of the RONNA G4 system – preliminary validation of 23 robotic frameless brain biopsies

**DOI:** 10.3325/cmj.2021.62.318

**Published:** 2021-08

**Authors:** Domagoj Dlaka, Marko Švaco, Darko Chudy, Bojan Jerbić, Bojan Šekoranja, Filip Šuligoj, Josip Vidaković, Fadi Almahariq, Dominik Romić, Marina Raguž

**Affiliations:** 1 Department of Neurosurgery, Dubrava University Hospital, Zagreb, Croatia; 2 Faculty of Mechanical Engineering and Naval Architecture, University of Zagreb, Zagreb, Croatia; 3 University of Zagreb School of Medicine, Zagreb, Croatia

## Abstract

**Aims:**

To report the outcomes of robot-assisted brain biopsies performed using a novel RONNA G4 system. The system was developed by a research group from the Faculty of Mechanical Engineering and Naval Architecture and a team of neurosurgeons from Dubrava University Hospital, University of Zagreb School of Medicine.

**Methods:**

This prospective study included 49 biopsies analyzed during one year: 23 robotic frameless and 26 frame-based Leksell stereotactic biopsies. We analyzed the presenting symptoms, tumor range and location, postoperative complications, pathohistological diagnosis, diagnostic yield, as well as operation and hospitalization duration. The target point error was calculated to assess the accuracy of the RONNA system.

**Results:**

No postoperative mortality, morbidity, or infections were observed. In the frameless robotic biopsy group, only one pathohistological diagnosis was inconclusive. Therefore, the diagnostic yield was 95.6% (22/23), similar to that of the framebased Leksell stereotactic biopsy group (95.1% or 25/26). The average target point error in the frameless robotic biopsy group was 2.15 ± 1.22 mm (range 0.39-5.85).

**Conclusion:**

The RONNA G4 robotic system is a safe and accurate tool for brain biopsy, although further research warrants a larger patient sample, comparison with other robotic systems, and a systematic analysis of the entry and target point errors.

Neurosurgery is one of the most demanding branches of medicine given the need for precision and limitations related to the anatomical area of interest and activity. Since the brain is a specific symmetrical organ enclosed in bone armor, precise localization of a target point within the skull is often challenging. Sophisticated technological innovations in neurosurgery, such as automated robot-assisted systems, offer a unique combination of precision, spatial accuracy, and dexterity. Several decades ago, the introduction of stereotactic frame into neurosurgery (1) established new standards in targeting and localization accuracy. The stereotactic frame is still widely used for brain biopsy procedures (2). The first robotic system used in neurosurgery was industrial robot PUMA 200 (3), after which a number of robotic systems have been developed (4-9). In comparison with humans, robotic systems have increased surgical accuracy, stability, non-fatigue, steadiness and endurance, accurate spatial positioning, quantitative analysis, extension of the visual and manual dexterity of neurosurgeons etc. Possible disadvantages include an inability to handle unexpected situations and required manual labor (10-12). Robotic systems are used in several neurosurgical procedures requiring exceptional spatial accuracy such as stereotactic biopsy, deep brain stimulation (DBS), stereoencephalography, external ventricular drainage, and endoscopy (6-8,13,14). Here, we present a new robotic neuronavigation system RONNA G4 developed by a research group from the Faculty of Mechanical Engineering and Naval Architecture, University of Zagreb, and a team of neurosurgeons from Dubrava University Hospital and the Zagreb University School of Medicine (15-18). Active research and development within the RONNA project started in 2010 (Figure 1) with an aim to create more precise and intuitive stereotactic neuronavigation procedures. The RONNA system has four generations. In the first generation, we developed a prototype localization device with a precisely calibrated camera and a laser triangulation sensor for the localization of a polymer reference localization marker (18). In 2012, the first-generation system was used in preclinical trials on phantoms. The second-generation system was a refined version of the first generation. As the master (navigation) robot we used KUKA Agilus KR6R900 sixx (16), which is still used in the fourth generation of the RONNA system. The mechanical pose repeatability of the Kuka Agilus KR6 R900 sixx robot arm with respect to the ISO 9283 is ±0.03 mm. In comparison, the mechanical accuracy of the ROSA system is ±0.1 mm (19).

**Figure 1:**
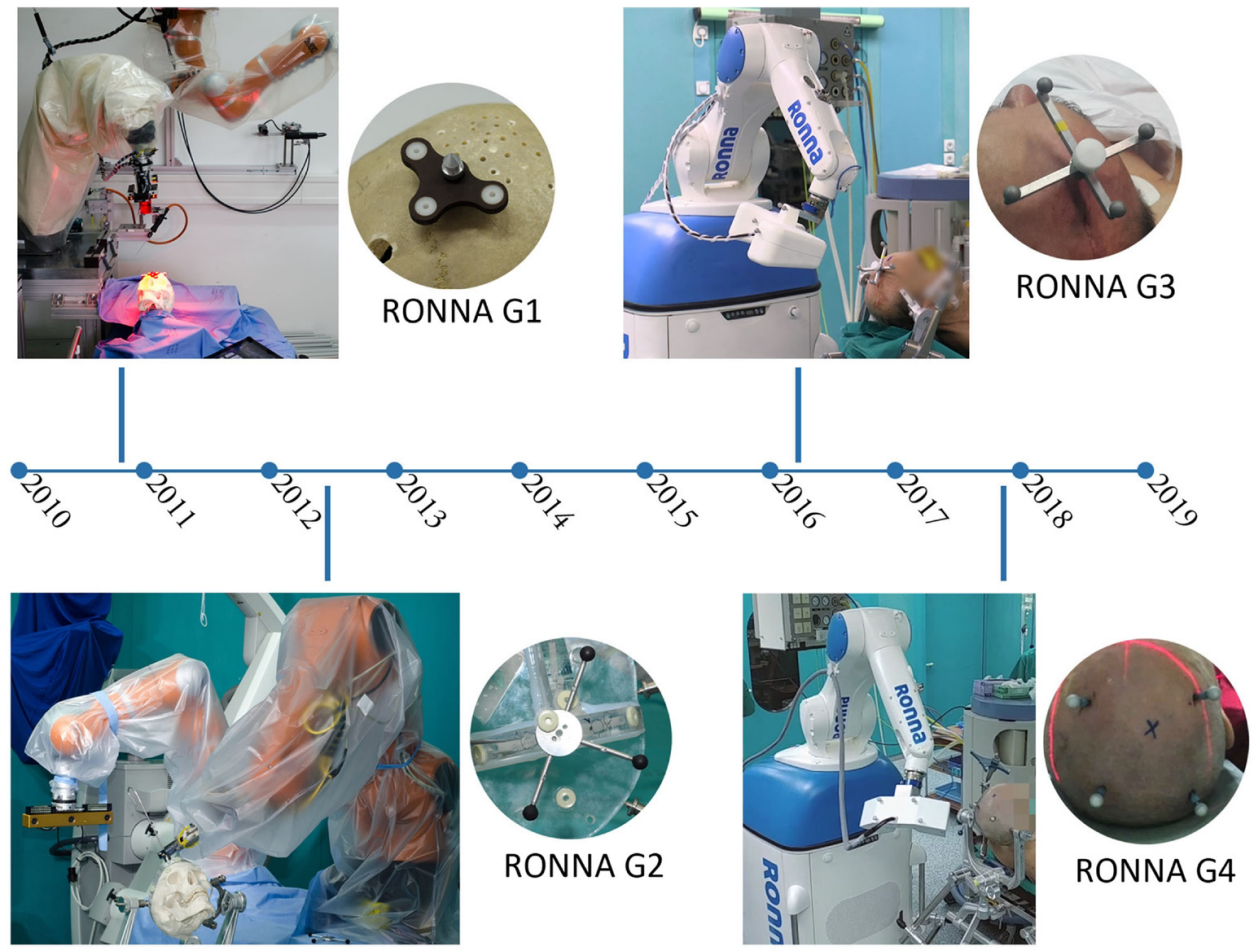
RONNA historical development.

On the second-generation localization marker, we developed a novel stereovision system for a precise localization of the spherical fiducials (20). The third-generation system, the RONNA G3, had a mobile platform with dedicated mechanical and electrical components (17,18). The RONNA G4 system includes multiple novel hardware and software improvements, which are described in detail by Jerbic et al (18). Currently, the RONNA G4 is clinically used as an important neurosurgical tool for preoperative planning and precise frameless neuronavigation (Figure 1). The first brain biopsy on an actual patient using the RONNA was performed at Dubrava University Hospital, Department for Neurosurgery in Zagreb, in May 2016 (17). Since then, the RONNA has been regularly used for stereotactic brain biopsies.

## RONNA G4 workflow

The RONNA G4, is currently in the clinical trial phase, and numerous laboratory tests and extensive preclinical trials were performed before clinical application (15,16,21).

**Figure 2:**
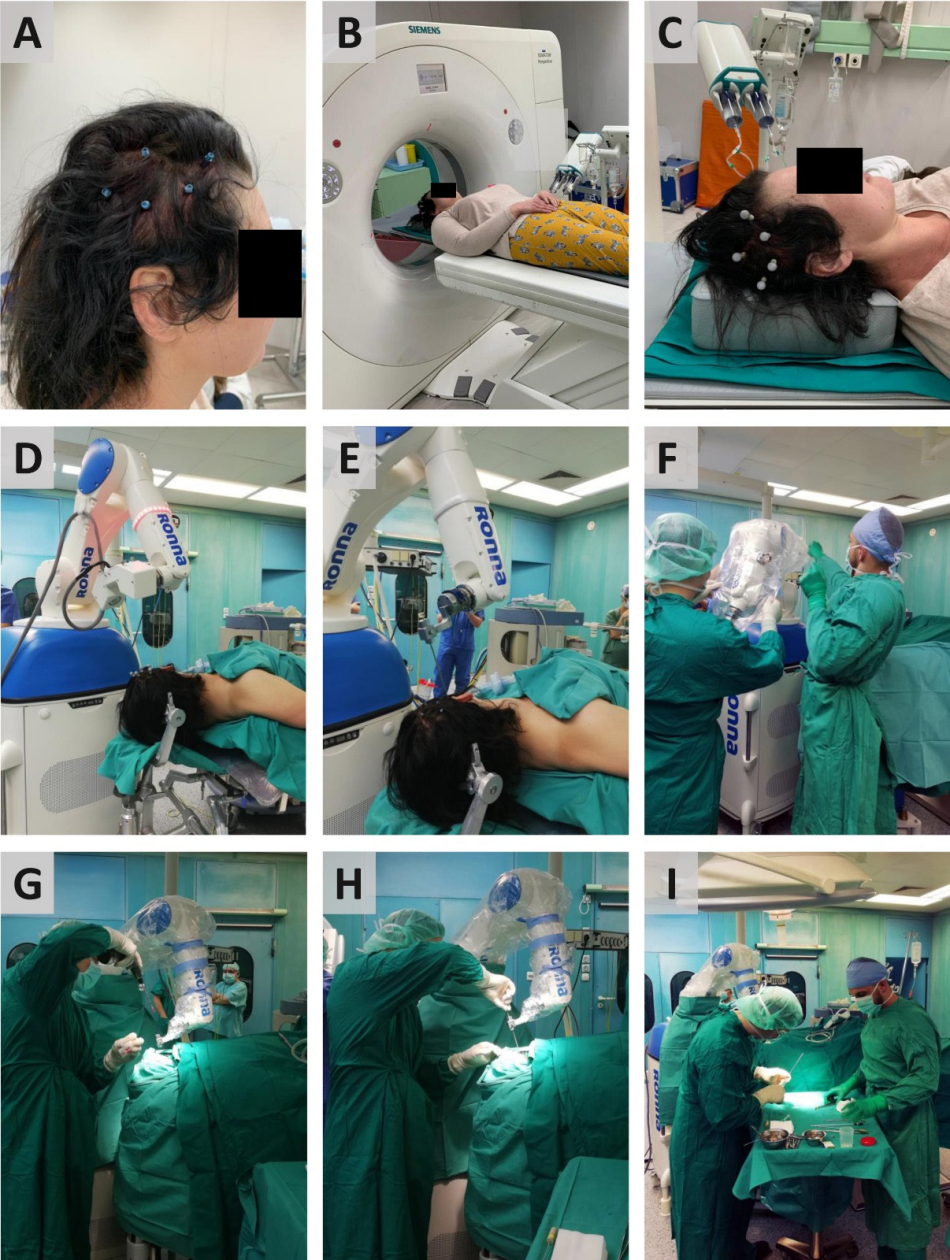
RONNA G4 surgical workflow. Before operation, the bone-attached screws are fixed to the patient’s head under local anesthesia (**A**). The patient undergoes a contrast-enhanced preoperative computed tomography and magnetic resonance imaging according to a standard head protocol (B,C). In the preparation phase, the robot is positioned in an optimal position near the patient. An optical tracking system is used to coarsely position the robot with respect to the patient (**D**). The trajectory is visualized using a non-sterile probe (**E**). In the operation phase, after anesthesia induction, the operating field is prepared, and a sterile cover is put on the patient and the robot (**F**). Using the RONNAplan, the neurosurgeon selects the preoperatively planned trajectory following the tool guide positioning by the robot. Skin incision and burr hole drilling are performed manually by the neurosurgeon (**G**). A biopsy needle is advanced manually by the neurosurgeon to the target point in order to obtain the tissue for pathohistological analysis (H, I).

The clinical procedure is performed in three stages (Figure 2). The procedure starts with the preoperative phase. Specially developed self-drilling and self-tapping screws are attached to the cranial bone under local anesthesia to allow precise localization and navigation. For frameless robotic system biopsies, screws are inserted percutaneously while the patient lies comfortably in his or her bed. In contrast, frame mounting requires a forced sitting position and patient's collaboration to keep his or her head still. Feeling the frame tightening around the head may cause considerable discomfort to the patient. In addition, the frame is carried until the end of biopsy, while the percutaneous screws mostly cannot be seen or felt. Several articles (22,23) report on better tolerance of percutaneous screw placement in patients undergoing frameless robotic system biopsies, supporting our protocol (22,23).

After screw placement, the patient undergoes contrast-enhanced preoperative computed tomography (CT) imaging (Somatom Emotion®, Siemens, Erlangen, Germany) according to our standard head protocol with the following parameters: gantry rotation time 0.6 s, helical scanning, detector collimation 16 mm ×0.6 mm, slice-width 0.75 mm, reconstruction increment 0.7 mm, image matrix 512 × 512 with a voxel size of 0.5 mm ×0.5 mm ×0.7 mm, no gantry. After scanning, the images are imported into the operation planning software (RONNAplan) integrated as a plugin for the Osirix MD medical certified software (Pixmeo, Sa'rl, Switzerland). The RONNAplan allows the automatic localization of spherical fiducial markers using our novel algorithm, as previously described (24). The software is non-hardware dependent and can be used as a plugin on any computer running Osirix MD. This allows the neurosurgeon to plan the operative trajectories in a decentralized fashion even a day before the surgical procedure, without the need for a special planning station, making the planning system scalable (Figure 2A-C). The final operative plan is transferred to the RONNA G4 control computer, where it is used for navigation in the operating theater.

The first phase is followed by a preparation phase. After positioning the patient in the operating theater, our novel robot positioning algorithm (25) calculates the optimal position of the robot with respect to the patient. The medical staff manually positions the robot base given the feedback from the global optical tracking system and visual instructions from the RONNAplan software module (Figure 2 D,E).

In the operation phase, after anesthesia induction, the operating field is prepared, and the patient and the robot are covered with a sterile draping. Using the RONNAplan, the neurosurgeon selects the preoperatively planned trajectory following the tool guide positioning by the robot. We developed an intuitive human-robot software interface for controlling intraoperative robot actions. The robot can be easily navigated and positioned in multiple positions, such as the main biopsy position, multiple staged-biopsy positions, drilling position, etc, and be adjusted to different depths of the same trajectory. In the current phase of the clinical trials, the system is used for brain-tissue biopsies. The robot uses a sterile tool holder for a twist drill and a biopsy needle (diameters: 3.2 mm and 2.5 mm, respectively). Skin incision and burr hole drilling are performed manually by the neurosurgeon. After the opening and electrocoagulation of the dura, a biopsy needle (diameter 2.5 mm) is advanced manually by the neurosurgeon to the target point. If needed, the neurosurgeon commands the robot a few millimeters deeper and/or superficial with respect to the target point to perform a staged biopsy. When the biopsy is completed at the target point, the robot retracts to its home position or repositions the tool guide at another target if multiple biopsy sites are planned (Figure 2 F-I).

The aim of our study was to assess efficacy and safety of robotic frameless and stereotactic framebased biopsies.

## Patients and methods

This prospective study involved two patient groups. In the first group, the brain biopsies were performed with the RONNA G4 robotic system, and in second group with a framebased stereotactic approach with a Leksell frame (Elekta AB, Stockholm, Sweden). No clinical or other criteria make a patient more eligible for robotic or stereotactic brain biopsy. Both procedures have the same indications; stereotactic biopsy should be performed whenever open surgery with at least bulk resection is not feasible or when the lesion requires only oncological treatment (26,27). Since our research group is actively developing the RONNA system for robotic frameless brain biopsies, we performed robotic biopsies whenever possible. Framebased Leksell stereotactic biopsies were performed on all occasions when any part of the robotic system underwent regular maintenance and upgrade, or when an engineer was not available (the engineer presence was preferred in the first stages of development and clinical usage until the system reliability was shown and a learning curve was established). For example, when the robotic system was unavailable due to a software upgrade during a three-month period, we performed only framebased Leksell stereotactic biopsies. From February 2019 till February 2020, we performed 49 biopsies: 23 robotic and 26 framebased Leksell stereotactic biopsies. Every patient was informed about the biopsy type and the procedure details. Only one patient refused to participate; the patient was psychoorganically changed due to the tumor extent and localization. The included patients underwent full both preoperative and postoperative neuroradiological examinations (MSCT and MRI) used for additional analysis. All patients or their family members signed informed consent. The ethical approval was obtained from the Institutional Review Boards of Dubrava University Hospital and the Zagreb University School of Medicine.

## Results

**Figure 3:**
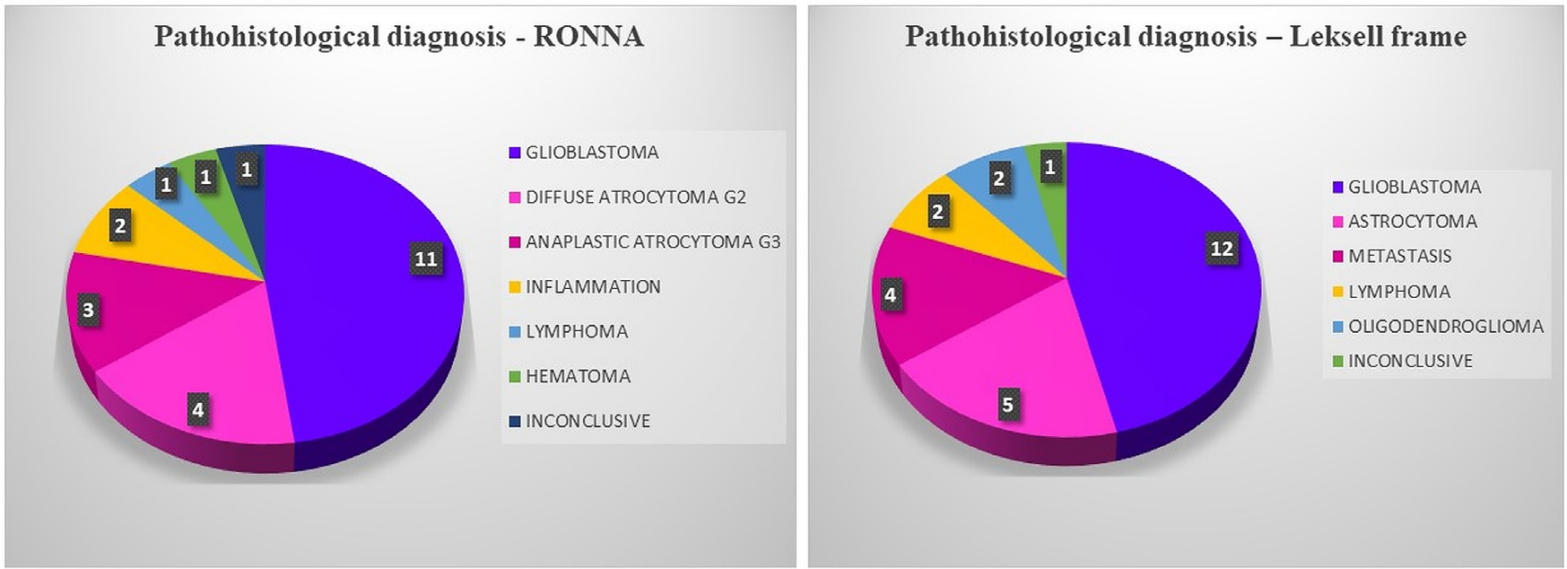
Results of the pathohistological analysis for the tumor tissue obtained using the RONNA G4 frameless brain biopsy and framebased stereotactic brain biopsy.

**Figure 4:**
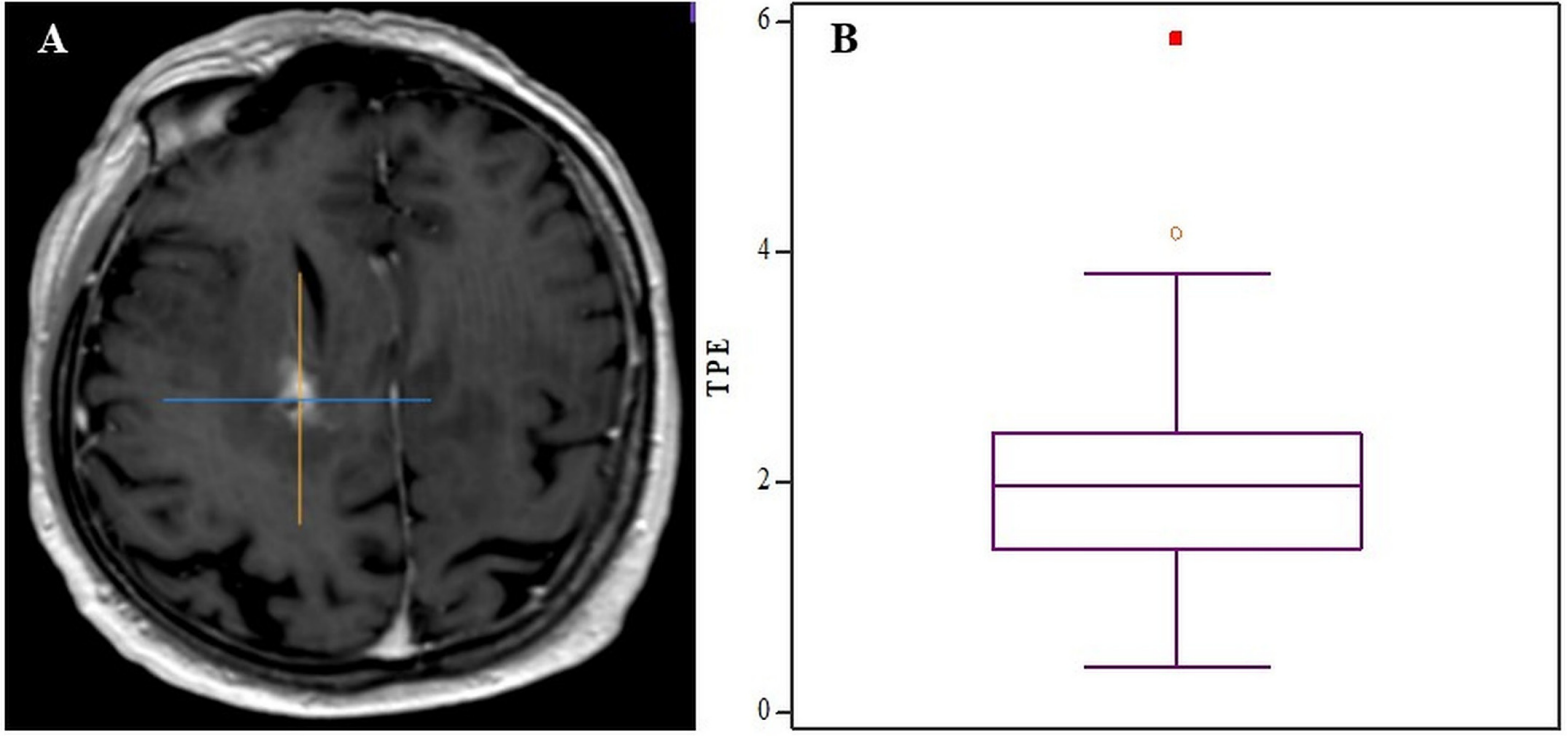
The methodology used for target point error measurement; tissue defect of tumor sampling site superimposed with a preoperative plan (**A**). A range of measured target point error values presented a mean ± standard deviation (**B**).

The robotic frameless brain biopsy group consisted of 23 patients (7 women), and the framebased Leksell stereotactic brain biopsy group consisted of 26 patients (12 women). In the frameless robotic biopsy group, the mean age of female patients was 64 ± 15.65 years (range 42-82 years), while that of the male patients was 56 ± 14.41 years (range 28-79 years). In framebased Leksell biopsy group, the mean age of female patients was 60 ± 10.10 years (range 50-80 years), while that of the male patients was 59.5 ± 15.09 years (range 24-79 years). None of the enrolled patients experienced postoperative mortality, morbidity, or infections. Presenting symptoms, tumor range, postoperative complications, and lesion locations, were similar in both groups (Table 1), stressing possible changes in patients’ presentation (Table 2). In the frameless robotic biopsy group, only one pathohistological diagnosis was inconclusive, with the diagnostic yield of 95.6% (22/23), similar to the framebased Leksell stereotactic biopsy group (96.1% or 25/26) (Figure 3). To calculate the target point error (TPE) and to quantify the accuracy of the RONNA system, we used a multimodal rigid body registration algorithm to register the postoperative MRI scan with the preoperative CT scan. TPE was measured on the registered MRI scan at visible deformations that were closest to the target point within the brain or the tumor. The distance from the planned trajectory and the postoperative defect on the MRI was calculated as a Euclidian distance, which gave the lateral error of the brain biopsy procedure (Figure 4). The average TPE in group I was 2.15 ± 1.22 (range 0.39-5.85) (Figure 4). Additionally, the duration of the operation, ie, the time from the moment the patient entered the operating theater until the operative procedure ended was 69.3 ± 20.3 min (range 41-128 min) in the frameless robotic biopsy group, and 38 ± 12.28 (range 21-57 min) in the framebased Leksell stereotactic biopsy group. The average hospital stay was 4.57 ± 1.08 days (range 3-6 days) in the frameless robotic biopsy group, and 6.5 ± 6.07 days (range 2-30 days) in the framebased Leksell stereotactic biopsy group. In the robotic frameless system group, complications occurred in three patients. One patient with a parietooccipital tumor, size 30 mm, experienced intracerebral hemorrhage without neurological worsening. Two patients experienced pneumocephalus, one with a frontal tumor size of 30-50 mm, without neurological worsening, and the other with a temporal tumor size of 30-50 mm, also without neurological worsening.

**Table 1:** Clinical characteristics of the included patients

	Robotic frameless biopsy			Classic stereotactic biopsy		
	female (n = 7)	male (n = 16)	all (n = 23)	female (n = 12)	male (n = 14)	all (n = 26)
Presenting symptoms						
motor (hemiparesis/monoparesis)	1/0	4/3	8 (5/3)	1/1	4/0	5/1
vertigo/ataxia		2/2	4 (2/2)	2	2	4
symptomatic epileptic seizures	1	3	4		2	2
headache		5	5	4	2	6
dysphasia	2	2	4	2	3	5
altered behavior		9	9	4	2	6
asymptomatic, regular control MRI	2	1	3		1	1
Tumor size (mm)						
≤30	3	7	10	4	2	6
30-50	3	3	6	5	7	12
≥50	1	4	5	2	4	6
multicentric		2	2	1	1	2
Complications						
intracerebral hemorrhage		1	1	2	1	3
neurological worsening	-	-	-	-	-	-
pneumocephalus	1	1	2			

**Table 2:** The locations of pathohistological samples

Location	Robotic frameless biopsy			Classic stereotactic biopsy		
	female (n = 7)	male (n = 16)	all (n = 23)	female (n = 12)	male (n = 14)	all (n = 26)
Thalamus	1	4	5	1	1	2
Temporal lobe	1	4	5	2	3	5
Insula	1		1		1	1
Parietal lobe	2		2	2	3	5
Occipital lobe		2	2	1		1
Cerebellum		1	1			
Frontal lobe	2	4	6	2	3	5
Brainstem		1	1	1		1
Corpus callosum				2	2	4
Basal ganglia				1	1	1

## Discussion

The current study showed no difference in the efficacy, diagnostic yield, and complications between frameless robotic and framebased Leksell stereotactic biopsy systems.

**Figure 5:**
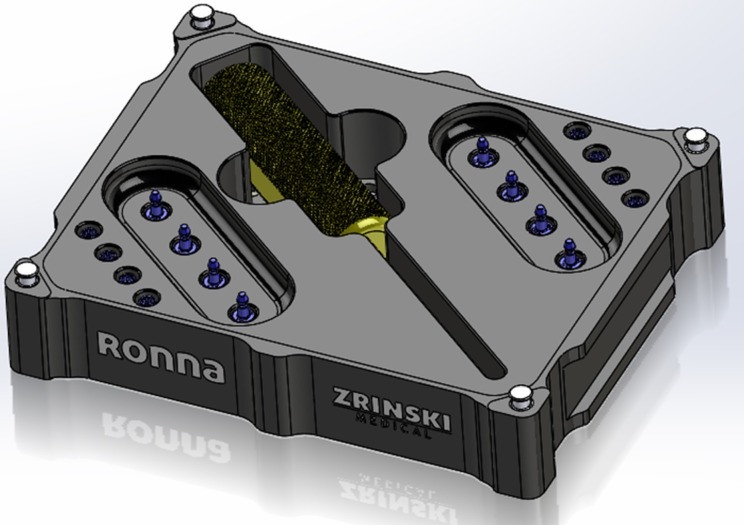
The RONNA bone screw set consisting of a milled aluminum instrument tray that holds eight screws, eight screw caps, and a specially designed screwdriver.

Surgical robotics is one of the most significant technological advances in neurosurgery. The fourth-generation RONNA has multiple new unique software and hardware features compared with other commercial or experimental robotic neuronavigation systems (4,5,28,29). The main feature are the freely distributed fiducial markers, ie, bone fiducials, which consist of a bone screw and a special screw cap with interchangeable spherical retroreflective fiducials. The RONNA G4 bone screw set consists of a milled aluminum instrument tray holding eight screws and eight screw caps, and a specially designed screwdriver (Eonex Medical, Trnovec, Croatia) (Figure 5). The whole set is sterilized before surgery in the autoclave. To minimize the effect of metallic artifacts in CT images, the screw caps are made of polyether ether ketone, a medical-grade polymer. The screw caps have a mechanical interface for standard medical retroreflective snap-fit spherical markers (NDI – Northern Digital Inc., Waterloo, Canada). When it comes to software features, RONNA G4 uses a new and improved algorithm for autonomous localization of fiducial markers in the image space (24), an algorithm for robot position planning with respect to the patient in the physical space (25), a novel pair-point correspondence algorithm for the patient registration from the image space to the physical space (30), and a novel method for determining an optimal robot localization strategy in the physical space (31). Other major features that differentiate the RONNA G4 from the current state of-the-art robotic neuronavigation systems are a specifically designed universal mobile platform (32) and a new and accurate non-contact localization system (18). The universal mobile platform can be used for different robot arms and is not limited to a unique robot model. The high-precision non-contact localization system RONNAstereo is used for accurate patient localization in the image space. For safety reasons, RONNA G4 has an uninterruptible power supply and 20-minute autonomy in case of power failure. It is also equipped with numerous built-in software and hardware safety measures. A speed limiter is used for all linear and joint movements to limit speeds to 30 mm/s or 5°/s. An emergency stop button (manual shutoff button) is incorporated both as a hardware and software option. All robot motions are planned before the execution, and collisions between the robot, its tools, and the patient are continuously checked.

Conventional frame-based procedures still represent the gold standard for brain biopsies. Although stereotactic frames such as Leksell, Cosman-Brown-Wells (Integra, Mayfield Cranial Stabilization System, Plainsboro, NJ, USA), and/or other frames are widely used (2), frameless biopsy has several advantages over stereotactic frame-based biopsies. One of the main advantages is less patient discomfort. Furthermore, frameless biopsies allow making preoperative scans a day before surgery, enabling a more flexible planning strategy. More time is available for a detailed operative plan analysis, allowing the planning of better trajectories. An advantage of the RONNA G4 robotic neuronavigation system and a head holder (such as a Mayfield clamp *–* Integra, Mayfield Cranial Stabilization System) in a frameless setup allows the creation of both lateral and posterior trajectories without hardware limitations, which is not the case in frame-based biopsies (17). In addition, frameless systems allow multiple trajectory planning and switching between trajectories within seconds, thus simplifying and speeding-up the procedure for multiple biopsy locations.

Several points should be discussed when comparing our study with previously published frame-based, frameless, and robotic biopsy studies (2,5,28,29,33). In other studies, the sample size varied from fewer than ten to more than 200 patients. Thus, we believe our preliminary results from a sample of 23 patients to be valid, considerable, and comparable with other studies in the field of stereotactic brain biopsies. In frame-based and frameless biopsy studies, the mortality ranged from around 1% to 4% and morbidity from 1% to more than 20%, and in robotic brain biopsies the mortality and morbidity rates were less than 10%. In our study, no postoperative mortality or morbidity were observed. Complications included intracerebral hemorrhage in only one patient and pneumocephalus in two patients, visible on postoperative CT and/or MRI scan, without neurological worsening. In the available frame-based and frameless biopsy studies, both hemorrhage and neurological deficit occurred in 1% to 20% of patients. In robotic brain biopsies, hemorrhage and neurological deficit occurred in approximately 4% to 28% of patients. The average procedure time ranges from 56.3 ± 23.6 to 185 ± 6 min for frameless biopsy studies and 54.2 ± 31.9 to 149 ± 32 min for frame-based studies, while the average operation duration in our study was 71 ± 20.7 min. Still, we believe that the average procedure time will shorten as neurosurgeons become more experienced. The diagnostic yield in our sample was 95.6% (22/23). In the literature, the diagnostic yield for various frameless biopsy cohorts ranged from 86.6% to 100%, for frame-based biopsy cohorts it ranged from 84% to 100%, and for robotic brain biopsies it ranged from 75% to 100% (2,5,28,29,33).

**Figure 6:**
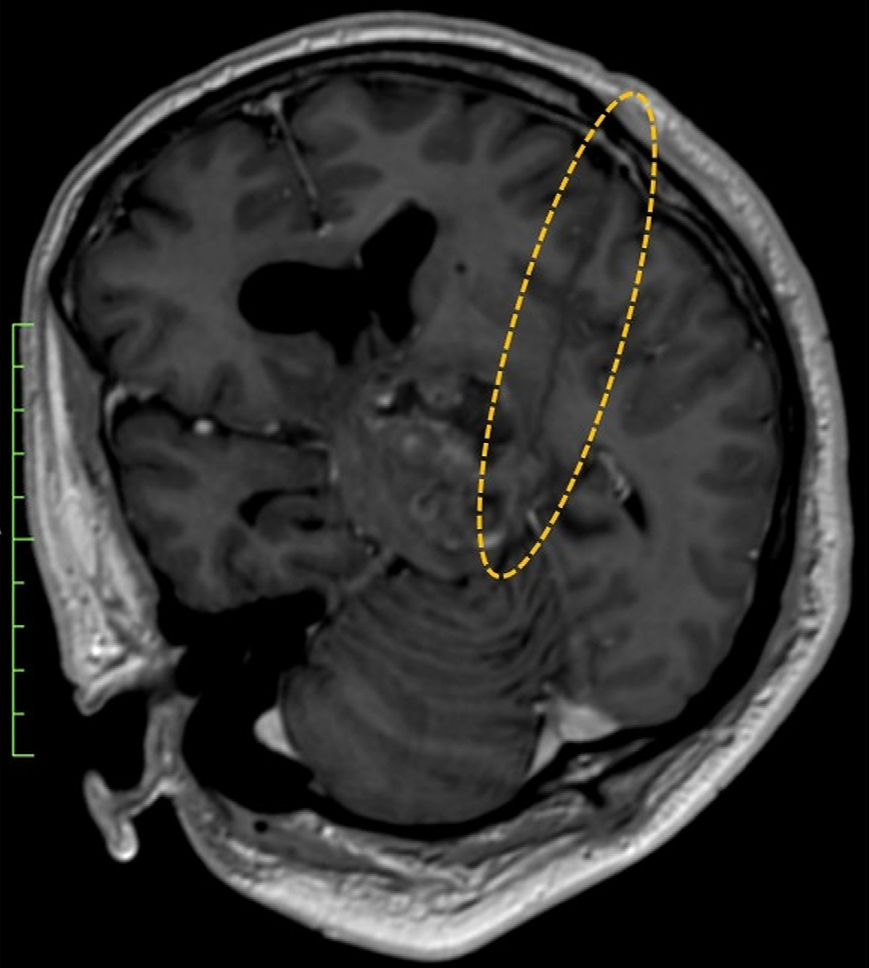
Trajectory cross-section of an inconclusive frameless robotic biopsy presenting target point on the tumor margin.

The single inconclusive sample was located at the level of the crura cerebri and mesencephalon. Usually, tissue samples obtained during brain biopsies are not sent for intraoperative pathohistological diagnosis. The reasons are short operation duration, a few small tissue samples obtained during biopsy (tissue cylinder), and the time needed to perform intraoperative pathological analysis. Tissue sampling during biopsy may lead to malignant tumor bleeding causing intracerebral hemorrhage in 5% to more than 20% of the cases (34). Thus, to avoid possible complications when performing deep-seated brain tumor biopsies with neuroradiological signs of malignancies, especially at the level of the crura cerebri as was the case in this patient, the target for tissue sampling is set on the tumor margin. We repeated the biopsy with Leksell frame; the pathohistological diagnosis was glioblastoma, and the patient underwent oncological treatment. On the repeated biopsy, based on our previous experience, we planned a riskier target point in the middle of the tumor since pathohistological diagnosis was mandatory for oncological treatment, which was the only treatment option for the patient (26,27). In addition, postoperative MRI did not show mistargeting (Figure 6). The obtained tissue was the tumor-surrounding tissue, as we planned due to the above mentioned reasons. In addition, several factors, such as high coronal trajectory angle, a long trajectory, and especially minimal bending of the biopsy probe due to passing through the sulci, ie, tissues of different resistance, as well as the planned marginal area contributed to an unsuccessful pathohistological diagnosis.

Thus, the obtained RONNA G4 diagnostic yield values are comparable and higher than the average diagnostic yield in other studies (2,5,28,29,33). One of the challenges to the widespread acceptance of robotics in neurosurgery is the ability to train neurosurgeons to use these new technologies. An increase in neurosurgeon’s experience over time is associated with shorter procedure duration, length of postoperative hospitalization, and the incidence of complications. In neurosurgery, technical advancements will continue to improve the speed, accuracy, and tactile ability in computer-assisted surgeries. Completely autonomous neurosurgical procedures are still a long way off, but robotics is already progressively changing the face of neurosurgery.

Our results should be interpreted with several limitations in mind. First, the number of participants is relatively small, and the results need to be confirmed in a larger cohort. In addition, an in-depth accuracy system analysis with included errors of measurement should be conducted, as was previously described in the literature (2,4,5,8,22). All other factors that considerably affect the accuracy and diagnostic yield should be investigated, and *in vitro* studies should be conducted (35). Different planning software programs and platforms prevented us from comparing the accuracy measurement between robotic frameless and framebased Leksell stereotactic biopsies.

In conclusion, the RONNA G4 robotic system is a safe and accurate tool for performing brain biopsy. Further studies need to enroll a larger patient sample for comparison with other robotic systems, to systematically analyze the entry and target point errors, and to investigate a wider application of robotic systems (DBS, SEEG, catheter placement). As the research on robotic brain biopsies is limited, our prospective study adds to the knowledge on robotic stereotactic brain biopsies. We plan to perform more detailed studies on robotic frameless and framebased Leksell stereotactic biopsies and their application accuracy.
